# Everolimus Related Fulminant Hepatitis in Pancreatic Neuroendocrine Tumor With Liver Metastases: A Case Report and Literature Review

**DOI:** 10.3389/fendo.2021.639967

**Published:** 2021-04-01

**Authors:** Shih-Chun Chang, Chun-Yi Tsai, Keng-Hao Liu, Shang-yu Wang, Jun-Te Hsu, Ta-Sen Yeh, Chun-Nan Yeh

**Affiliations:** Department of General Surgery, Chang Gung Memorial Hospital at Linkou, Chang Gung University College of Medicine, Taoyuan, Taiwan

**Keywords:** acute hepatitis B flare-up, everolimus, fulminant hepatitis B, neuroendocrine tumor (NE tumor), pancreatic neuroendocrine tumor (pNET)

## Abstract

**Background:**

Everolimus, an immunosuppressant, is approved for the treatment of advanced renal cell carcinoma, metastatic hormone receptor-positive breast cancer, and pancreatic neuroendocrine tumors (P-NETs) but has been reported to be related to hepatitis B reactivation. Here, we present the first case of fatal fulminant hepatitis B reactivation in a man with P-NET accompanied by multiple liver metastases who received everolimus and octreotide long-acting repeatable (LAR).

**Case Presentation:**

A 45-year-old male had a history of chronic hepatitis B infection. He was found to have a complicated liver cyst incidentally, and then he underwent biopsy, which disclosed a grade 2 neuroendocrine tumor (NET). Subsequent MRI of the abdomen and PET revealed a solid mass at the pancreatic tail with numerous liver tumors favoring metastases and peripancreatic lymph node metastases. Transarterial chemoembolization (TACE) of the right lobe of the liver was performed, and he started to take 5 mg everolimus twice a day and 20 mg octreotide LAR every month 8 days after the 1^st^ TACE. No hepatitis B virus (HBV) prophylaxis treatment was administered. He then underwent laparoscopic distal pancreatectomy and splenectomy three and half months after the initial treatment of everolimus. He continued everolimus 5 mg twice a day and octreotide 20 mg every month after the operation. Three months later, hepatic failure occurred due to acute hepatitis B flare-up–related fulminant hepatic failure since other possible causes of hepatic failure were excluded. Five days after hepatic failure presented, hepatic failure was apparent, and pulseless ventricular tachycardia occurred. The patient expired after failed resuscitation.

**Conclusion:**

A literature review of everolimus-related hepatitis B reactivation was conducted. In P-NET patients with chronic hepatitis B who will undergo everolimus treatment, HBV prophylaxis should be considered since fatal hepatitis B reactivation might occur under rare conditions.

## Background

Everolimus, at type of mammalian target of rapamycin (mTOR) inhibitor, is approved for the treatment of advanced renal cell carcinoma, metastatic hormone receptor-positive breast cancer, and pancreatic neuroendocrine tumors (P-NETs). However, as an immunosuppressant, everolimus has been reported to be related to hepatitis B reactivation. Previous publications have proposed fatal hepatitis B reactivation in patients receiving everolimus for metastatic breast cancer and advanced renal cell carcinoma. Here, we present the first case of fatal fulminant hepatitis B reactivation in a man with P-NET accompanied by multiple liver metastases who received everolimus and octreotide long-acting repeatable (LAR).

## Case Report

A 45-year-old male had a history of chronic hepatitis B infection and hypertension and a mild increase in glucose levels. He had been regularly followed up in a gastrointestinal outpatient clinic for chronic hepatitis B infection. Half a year before admission, a complicated liver cyst was incidentally found on liver sonography in another hospital, where computed tomography (CT)-guided biopsy of the complicated liver cyst was performed. The pathology report revealed grade 2 neuroendocrine tumors (NETs) with a mitotic figure of 7 in 10 high-power fields. Immunohistochemical staining confirmed the diagnosis of NETs with positive chromogranin A, synaptophysin, and CD 56 staining. The lesion was negative for CK-7 and hepatocellular carcinoma markers, including Hepar-1, arginase-1, and glypican-3. Metastatic NET was considered. During this period, there was no abdominal pain, flushing, or diarrhea but mild cold sweating.

**Figure 1:**
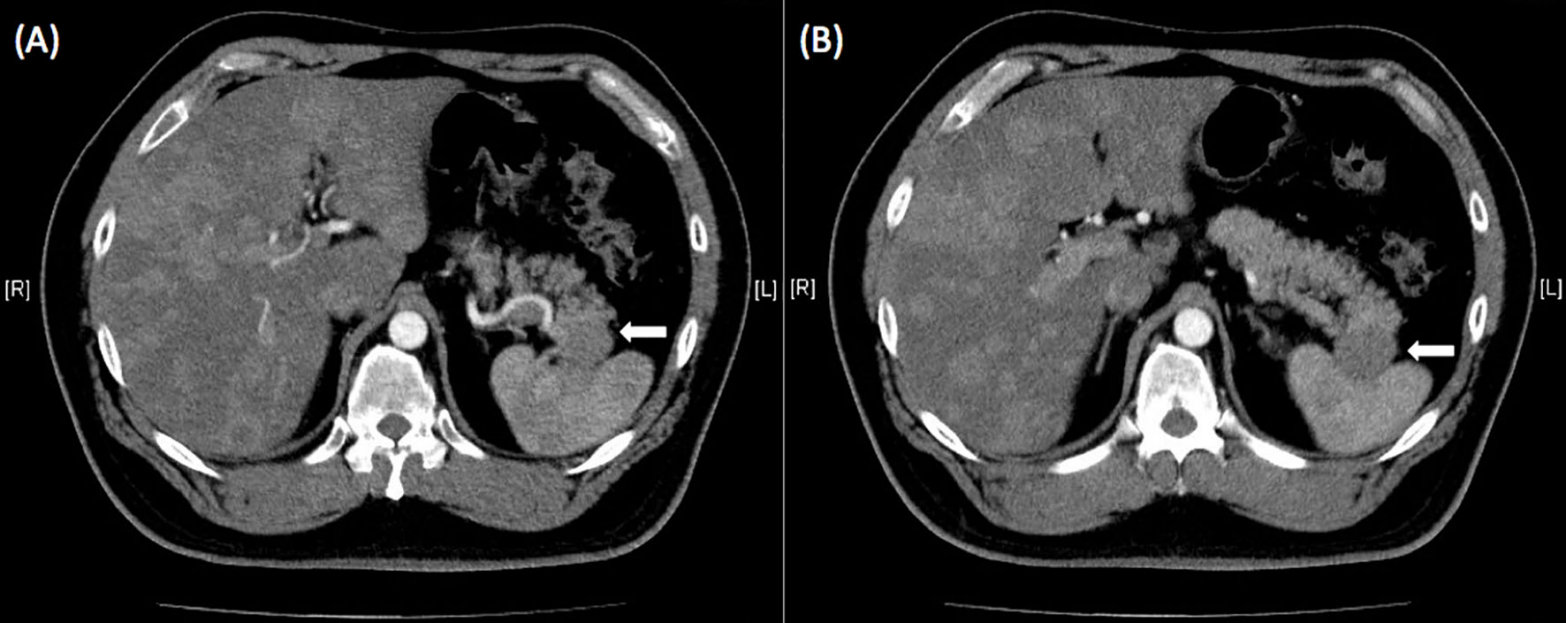
**(A)** Abdominal computed tomography showed a pancreatic body tumor approximately 1.5 cm in size associated with liver tumors in the right and left lobes. Adrenal glands were normal without enlargement. **(B)** A suspected tumor at the pancreatic tail was disclosed on enhanced abdominal computed tomography (white arrow).

**Figure 2:**
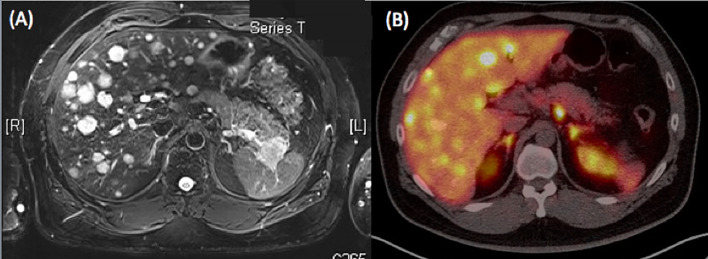
**(A)** MRI of the abdomen revealed a solid mass at the pancreatic tail with numerous liver tumors favoring metastases. The pancreatic body tumor was a cyst. **(B)** PET with MRI also showed peripancreatic lymph node metastases.

He was then hospitalized in our hospital. The hemogram and biochemistry investigations were normal, including serum carbohydrate antigen 19-9 (CA 19-9) levels (<0.6 IU/L, normal: <37 IU/L), carcinoembryonic antigen (CEA) levels (1.36 ng/mL, normal: <5 ng/mL), and alpha-fetoprotein levels (3.8 ng/mL, normal: <9 ng/mL). Serum chromogranin A levels were elevated (119.80 ng/mL, normal: <101.9 ng/mL), but adrenal function (cortisol 12.87 µg/dL, normal: 7-9AM 4.2-22.4 µg/dL, 3-5PM 3.1-16.7 µg/dL; adrenocorticotropic hormone (ACTH) 35.10 pg/mL, normal: 7.2-63.3 pg/mL), gastrin levels (44.2 pg/mL, normal: 28-185 pg/mL), and C-peptide levels (4.5 ng/mL, 1.1-4.4 ng/mL) were all within the normal range, as were urinary vanillylmandelic acid (VMA) (8.1 mg/day, normal: 1.9-9.8 mg/day) and 5-hydroxyindoleacetic acid (5-HIAA) (4.8 mg/day, normal: 2-6 mg/day) levels. Hepatitis B surface antigen (HBsAg) (7069.00, nonreactive: <0.9, equivocal: 0.9-10, reactive: >10), anti-HBs antibody (528.50 IU/L, nonreactive: <10), and anti-HBc antibody (0.005, non-reactive: >1.0) were all reactive but nonreactive to anti-hepatitis C virus (HCV) antibody. Subsequent abdominal CT showed a pancreatic body cystic tumor approximately 1.5 cm in size (**Figure 1A**) and a suspected pancreatic tail tumor associated with liver tumors in the right and left lobes (**Figure 1B**). Magnetic resonance imaging (MRI) of the abdomen revealed a solid mass at the pancreatic tail with numerous liver tumors favoring metastases. The pancreatic body tumor was a cyst (**Figure 2A**). Positron emission tomography (FDG-PET) with MRI also showed peripancreatic lymph node metastases (**Figure 2B**). Transarterial chemoembolization (TACE) of the right lobe of the liver was performed during this admission. He was discharged 3 days after TACE.

**Figure 3:**
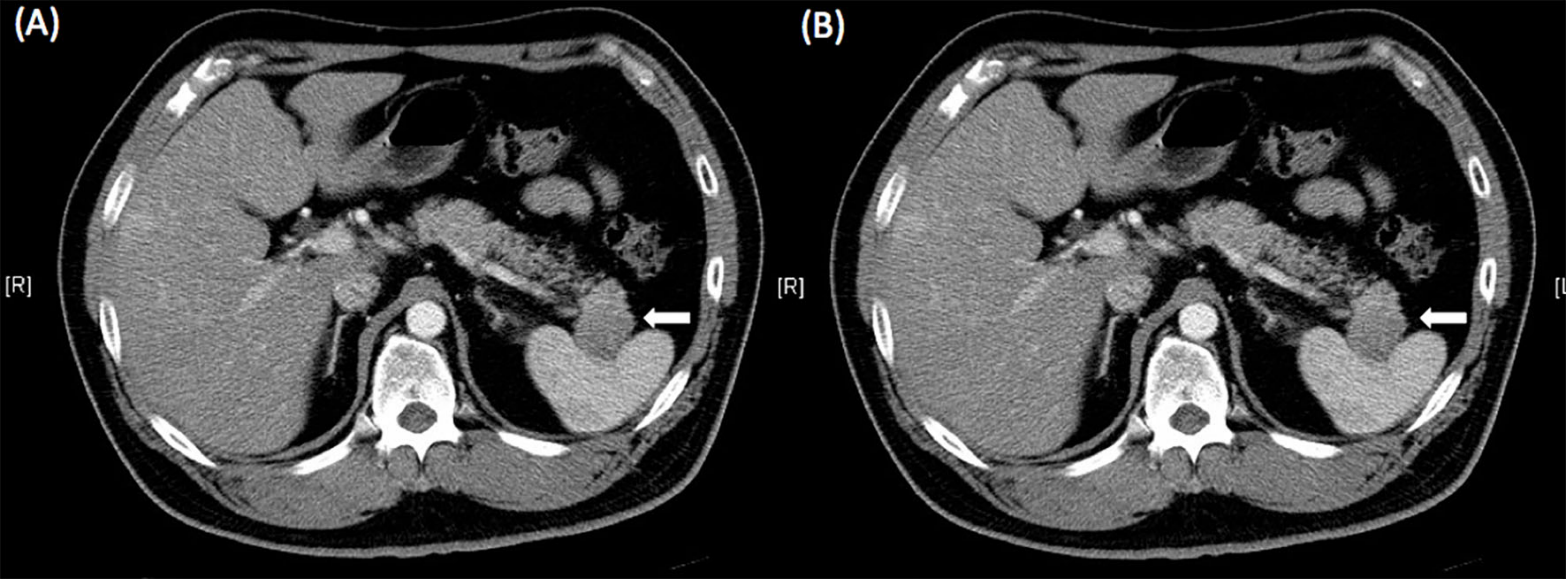
Abdominal computed tomography after 3 months of treatment with everolimus showed stationary pancreatic neuroendocrine tumors (**A**, white arrow) but decreasing liver metastasis nodule numbers **(B)**, suggesting a partial response to everolimus.

**Figure 4:**
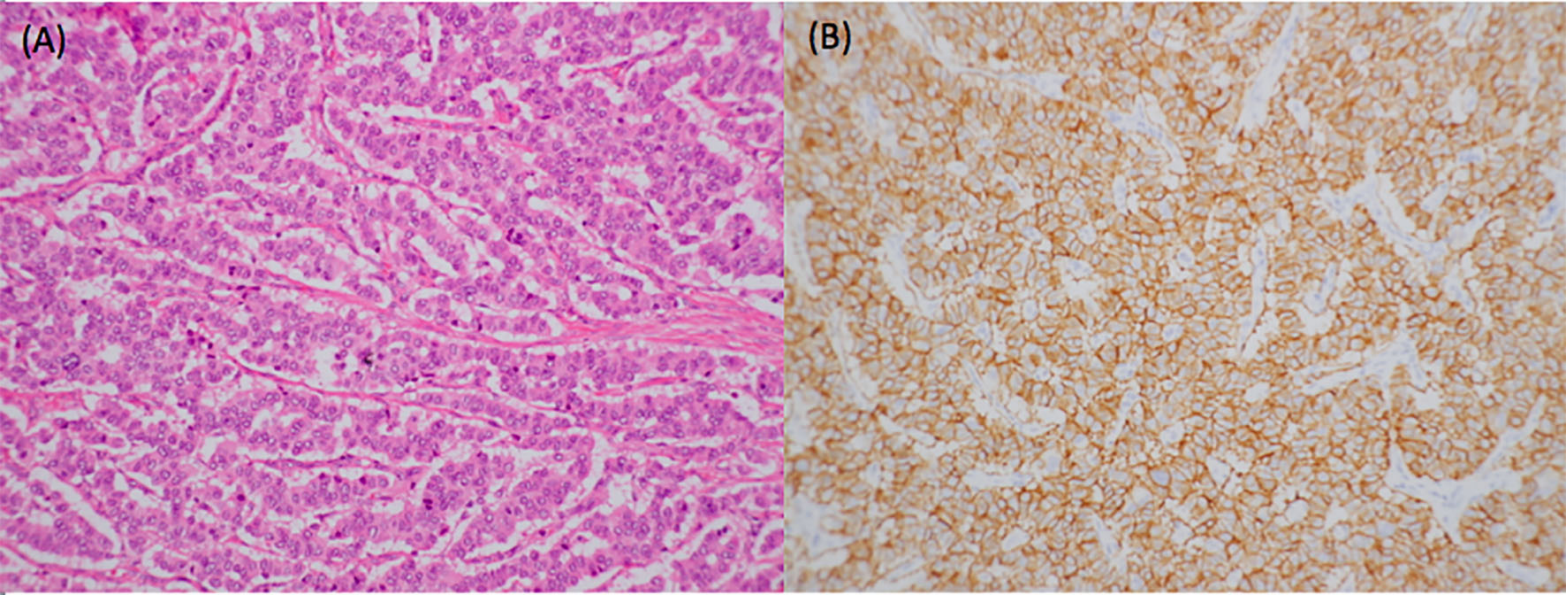
Pathology from distal pancreatectomy showed a neuroendocrine tumor with numerous mitotic figures **(A)**. Immunohistochemical analysis revealed that tumor cells were all positive for CD56, chromogranin A, synaptophysin, and beta-catenin on the membrane but negative for insulin. Ki-67 index was 15%. Therefore, it was a grade 2 neuroendocrine tumor **(B)**.

He started to take 5 mg everolimus twice a day and 20 mg octreotide LAR every 28 days, starting from 8 days after the 1^st^ TACE. No hepatitis B virus (HBV) prophylaxis treatment was administered. After he took everolimus for three months and octreotide 3 times, and subsequent abdominal CT showed stationary pancreatic NETs but decreased liver metastasis nodule numbers, suggesting partial response to concurrent everolimus **(** **Figures 3A, B**). Therefore, he underwent laparoscopic distal pancreatectomy and splenectomy three and half months after the initial treatment of everolimus. The pathology report revealed grade 2 NETs of the pancreas with direct invasion of the spleen. Immunohistochemical analysis revealed that tumor cells were positive for CD56, chromogranin A, synaptophysin and beta-catenin on the membrane and negative for insulin. Ki-67 index was 15% **(** **Figures 4A, B**). The postoperative course was uneventful, and he was discharged 7 days after the surgery.

He continued everolimus 5 mg twice a day and octreotide 20 mg every 28 days after the operation. No HBV prophylaxis treatment was administered as before. Two and half months after the surgery, he underwent abdominal CT for follow-up, which disclosed no evidence of local recurrence at the pancreas and regression of the liver tumor where previous TACE was performed; the other liver metastases appeared as stable disease.

Approximately 2 weeks after the last abdominal CT, he had general weakness with an icteric look and then coma. He visited another hospital abroad, and was initially hospitalized there; hepatic failure progressed there. Four days later, he was transferred back to our hospital and admitted to the intensive care unit (ICU). The hemogram and biochemistry study showed coagulopathy but no thrombocytopenia (international normalized ratio (INR) 2.9, platelet count 22,6000/µL), jaundice (total bilirubin 11.1 mg/dL, direct bilirubin 6.9 mg/dL), abnormal liver function test (aspartate aminotransferase (AST) 824 U/L, alanine aminotransferase (ALT) 636 U/L), azotemia (blood urea nitrogen (BUN) 21.2 mg/dL, creatinine 5.87 mg/dL) or elevation in ammonia level (408 µg/dL). The HBV DNA level was 1.832509 million cps/ml, and the anti-hepatitis B e antibody was reactive, so entecavir was prescribed. Anti-hepatitis C antibody, CMV-IgM, HIV antigen, EB-VCA IgM, and RPR were all nonreactive. Acute hepatitis B flare-up–related fulminant hepatic failure was diagnosed since other possible causes of hepatic failure were excluded. However, general tonic clonic seizures occurred in the ICU, but brain CT showed no obvious lesions. Five days after hepatic failure was apparent, pulseless ventricular tachycardia occurred. The patient expired after failed resuscitation.

## Discussion

Everolimus is approved as a single agent for the treatment of advanced renal cell carcinoma and P-NETs and as combination treatment with exemestane for the treatment of hormone receptor-positive breast cancer (1–3). Pavel et al. published the randomized, double-blind, placebo-controlled, phase 3 RADIANT-2 study in which median progression-free survival (PFS) improved for 5.1 months with everolimus plus octreotide LAR compared with placebo plus octreotide LAR in patients with low- or intermediate-grade advanced NETs and a history of carcinoid symptoms (4). Despite no significant improvement in PFS, the latest result of the final overall survival from the RADIANT-2 study was positive with a hazard ratio of 1.08 (5), and everolimus plus octreotide LAR was still considered an effective approach for these patients. This finding was supported by our patient having stable disease of the original P-NET tumor but significant improvement in liver metastases, in which the tumor burden seemed decreased under the control of everolimus plus LAR. However, acute flare-up of hepatitis B leading to fulminant hepatic failure occurred when everolimus plus LAR was used for 5.5 months. One out of 204 patients in the RADIANT-2 trial developed fatal HBV reactivation (4). A similar event has been reported in a patient with metastatic breast cancer who received treatment with everolimus plus exemestane for a 15-day period (6). Everolimus-related acute hepatitis reactivation has also been reported in renal cell carcinoma, in which one event occurred after a 3-month period of everolimus use and another after a 5-month period (7, 8). The latter patient died of fulminant hepatitis. **Table 1** summarizes the case reports on everolimus-related HBV reactivation. According to the literature review, this rare but fatal event seems to occur from 0.5 to 6 months after the start of use of everolimus. In breast cancer, fatal events seem to occur earlier (0.5 months and 3 months), and in renal cell carcinoma, they seem to occur later (both 5 months). The fatal event in our case tended to occur later (5.5 months), which is similar to that in renal cell carcinoma. However, since the number of case reports is still limited, the risk factors and the duration of HBV reactivation after everolimus use still need further investigation.

**Table 1:** Case reports about everolimus related HBV reactivation.

Year	Disease	Medicine used	Duration of treatment till HBV reactivation	Treatment of HBV reactivation	Outcome
2013 Sezgin et al. (7)	RCC with lung and axillary metastases	Everolimus 10mg daily then tapering to 5mg daily	5 months	Tenofovir	Resolved
2013, Shinta et al. (8)	RCC with lung metastases	Everolimus	5 months	Entecavir and steroid pulse therapy (methylprednisolone, 1000 mg/day for 3 days with gradual tapering)	Died
2013, Eleonora et al. (6)	Breast cancer with lung, bone, pancreas, intramuscular metastases	Everolimus 10mg daily+ exemestane 25mg daily	24 days	Tenofovir	Died
2016, Olivier et al. (3)	Breast cancer with bone metastases	Everolimus 10mg daily+ exemestane 25mg daily	3 months	Entecavir	Resolved
2021 Chang et al.	P-NET accompanied by multiple liver metastases	Everolimus 5mg twice a day + long acting octreotide 20mg every month	5.5 months	Entecavir	Died

RCC, Renal cell carcinoma.

Based on recent estimates, approximately 350 million people worldwide suffer from chronic hepatitis B infection (9). HBV reactivation is defined as a sudden and rapid increase in the HBV DNA level by at least 100-fold in patients with previously detectable HBV DNA or the reappearance of HBV DNA viremia in individuals who did not have viremia before the initiation of immunosuppressive or biological modifier therapy or cancer chemotherapy. Five stages have been proposed regarding HBV reactivation related to immunosuppressive or biological modifier therapy or cancer chemotherapy (9). HBV reactivation should particularly be paid attention to when people are exposed to cancer chemotherapy, immunosuppressive therapy, or biologic therapies for the management of malignancies or benign conditions, such as rheumatologic conditions, inflammatory bowel disease, dermatologic conditions, or solid-organ or bone marrow transplantation (9, 10). **Table 2** summarizes cytotoxic and immunosuppressive agents that have been reported to be related to HBV reactivation.

**Table 2:** Cytotoxic or immunosuppressive agents associated with HBV reactivation (6, 11).

Alkylating agents	Cyclophosphamide, Chlorambucil, Cisplatin, Temozolomide, Procarbazine
Alkaloids	Vincristine, Vinblastine
Antimetabolites	Cytarabine, Fluorouracil, Gemcitabine, Mercaptopurine, Methotrexate, Thioguanine
Monoclonal antibodies	Rituximab (anti-CD20) Alemtuzumab (anti-CD52) Mogamulizumab (anti CC-chemokine receptor 4) Anti-TNF-alpha (infliximab, adalimumab, golimumab, and certolizumab)
Other cytotoxic agents	Docetaxel, Etoposide, Fludarabine, Mitomycin, Bleomycin
Tyrosine kinase inhibitor (TKI)	Imatinib, Nilotinib, Dasatinib, Erlotinib, Ibrutinib
Other	Interferon

Everolimus is an mTOR inhibitor, and other similar medicines include rapamycin. The immunosuppressive properties of everolimus may predispose patients to opportunistic infections and/or the reactivation of previous infections. As expected, infective pneumonia and other bacterial and invasive fungal infections have been reported in patients treated with everolimus, as well as the reactivation of viral infections (7, 12), including hepatitis E virus (13–16). Another mTOR inhibitor, sirolimus, has also been reported to be associated with the reactivation of hepatitis B with octreiotide previously (17). The possible mechanism was that octreotide has been proposed to significantly reduced hepatic blood flow that decreased the liver metabolic activity in patients with hepatitis B surface antigen positive cirrhotic patients, which might be relevant to the reactivation of hepatitis B (18). In addition, somatostatin has been be hypothesized to be related to autocrine and paracrine regulatory role, and *via* neuro-endocrine modulation of the immune response, it might represent a direct regulatory relation between the nervous and immune system (19). Consequently, as an analogue of somatostatin, octreotide might play similar role. In contrast, several mechanisms have been proposed regarding the relationship between HBV inactivation, instead of activation, from mTOR inhibitors (20–22). Consequently, the mechanism by which fulminant hepatitis B originates from everolimus remains unclear.

Due to the increased risk of the reactivation of hepatitis B in patients who will receive immunosuppressive or cytotoxic therapies, many institutes have suggested screening before treatment is initiated, with at least HBsAg, anti-HBc, and anti-HBs (9, 10, 23–28); all those who are negative for HBsAg, anti-HBc, and anti-HBS should be vaccinated against HBV. Although no guidelines are available concerning the feasibility of antiviral prophylaxis combined with everolimus in treating P-NET or breast cancer, lamivudine, entecavir or tenofovir were suggested for anti-HBV prophylaxis in advanced renal cell carcinoma patients receiving everolimus (29). The duration of prophylaxis remains inconclusive. However, according to the European Association for the Study of the Liver (EASL) 2017 clinical practice guidelines, HBV prophylaxis should continue for at least 12 months and 18 months for rituximab-based regimens after the cessation of immunosuppressive treatment and discontinued only if the underlying disease is under remission. Close follow-up is also suggested, including liver function tests and HBV DNA during prophylaxis lasting for at least 12 months after antiviral agent withdrawal, since HBV reactivation might develop after antiviral agent discontinuation (26).

In conclusion, in P-NET patients who will receive everolimus plus octreotide LAR, HBV reactivation might occur, though the incidence is low. The duration of everolimus use for HBV reactivation is still inconclusive. The mechanism between everolimus and hepatitis B reactivation remains unclear. The protocol for HBV prophylaxis in everolimus is not well established. However, since fatal reactivation events have been reported in advanced renal cell carcinoma and metastatic breast cancer, clinicians should consider routine HBV screening and antiviral prophylaxis before everolimus therapy is initiated for P-NET patients receiving everolimus plus octreotide LAR.

## Data Availability Statement

The original contributions presented in the study are included in the article/supplementary material. Further inquiries can be directed to the corresponding author.

## Ethics Statement

Written informed consent was obtained from the patient’s family for the publication of this case report and any accompanying images.

## Author Contributions

C-YT, K-HL and S-YW: discussion and review about the organization of this article, and also deal with the pathology and image of this article. J-TH and T-SY: assist with the revised of article. All authors contributed to the article and approved the submitted version.

## Funding

This work was supported by the Chang Gung Medical Research Program, Taiwan.

## Conflict of Interest

The authors declare that the research was conducted in the absence of any commercial or financial relationships that could be construed as a potential conflict of interest.
